# Communication Difficulties Faced by Mothers of Neonates in Selected Neonatal Care Units

**DOI:** 10.7759/cureus.81799

**Published:** 2025-04-06

**Authors:** Shaila Mathew, Rohini P Dani

**Affiliations:** 1 Child Health Nursing, Bharati Vidyapeeth (Deemed to be University) College of Nursing, Sangli, IND

**Keywords:** assess, communication difficulties, mothers, neonatal care units, neonates

## Abstract

Introduction

For any mother, a child is very precious. The number of preterm babies, low-birth-weight babies weighing less than 2 kg, and Neonatal Intensive Care Unit (NICU) admissions is increasing day by day. It is a very stressful situation for the mother. Mothers face many difficulties while their baby is in the NICU, especially in communicating with health workers and getting answers to their concerns about their child’s condition.

Methodology

A quantitative approach with a descriptive design was used to conduct the study. A nonprobability purposive sampling technique was used to select the samples. Mothers whose neonates were admitted for a minimum of eight days were selected. The data collection tool had two sections: demographic variables and a scale for assessing the communication difficulties faced by the mothers, using a three-point Likert scale. Reliability of the tool was established using the split-half method. Frequency and percentage were calculated to assess the difficulties.

Result

The analysis revealed various aspects of communication difficulties at different levels. While the majority (48.75%) stated that explanations about special treatment were given before it was administered to the child, 28.75% said it was provided only sometimes. However, they were satisfied with the childcare, as the staff encouraged them to participate in their child's care. Regarding the tone of language, 25% stated that it was not appropriate. Mothers verbalized that the language used was simple to understand, and 66.25% said the information given was never confusing. Participants were happy with the visiting time, and 68.75% reported that sometimes instructions were given at an inappropriate time.

Conclusion

The study concluded that mothers faced difficulties due to the staff's approach, the explanations about their child's condition, and the counseling provided to the parents. Participants were somewhat afraid that expressing grievances might negatively affect their child's care. The study highlighted the need to improve professional practices, which can be achieved through in-service education.

## Introduction

Communication is a fundamental human interaction and a critical component of healthcare delivery. It is defined as the process of exchanging information, ideas, and emotions effectively between individuals. However, for individuals facing communication difficulties, including those with limited speech or challenges in understanding or expressing themselves, this process can be strenuous. In healthcare, particularly in high-stress environments like Neonatal Intensive Care Units (NICUs), effective communication becomes even more essential [1]. In the NICU, parents continually engage in communication with healthcare staff. Communication between parents and providers serves important clinical goals [2]. Communicating complex and large volumes of information during a perinatal crisis presents inherent challenges for both clinicians and families [3].

The World Health Organization (WHO) estimates that over 140 million babies are born globally each year, with many requiring intensive medical care shortly after birth. For mothers, the unexpected admission of their newborn to an NICU can be a deeply distressing experience. This situation is often compounded by communication barriers, especially for those who are illiterate or unfamiliar with medical terminology. These mothers face challenges in understanding their infant’s condition, asking questions, or engaging with healthcare staff, further adding to their emotional stress [4,5].

An infant’s admission to an NICU inevitably causes emotional stress for the parents. Communication between parents and NICU staff is an essential part of the support offered to the parents and can reduce their emotional stress. The aim of this study was to describe parents’ experiences of communication with NICU staff [6].

NICUs are designed to provide specialized care for critically ill neonates, but the technical nature of this environment can be overwhelming for parents. Effective communication between mothers and NICU staff is crucial to mitigate stress, foster understanding, and ensure active parental involvement in the infant’s care. However, studies reveal that mothers often encounter difficulties in receiving adequate attention from staff due to high workloads, time constraints, and the prioritization of the infant’s immediate medical needs over parental concerns. In some healthcare settings, the staff-to-patient ratio can exacerbate these challenges, leaving mothers feeling unsupported and isolated. An infant’s admission to an NICU causes inevitable stress to the parents. Communication between staff and parents can reduce this emotional stress [7].

Parents are dependent on the staff for their child's care and for controlling their emotional stress. A lack of communication leads to feelings of loneliness, the imagination of worst-case scenarios, and unnecessary frustration [3].

Neonates are admitted to the NICU primarily due to prematurity, low birth weight, Respiratory Distress Syndrome (RDS), and neonatal sepsis. Studies show that a quarter of total admissions result in death each year. These factors contribute significantly to parental stress. Caretakers need to share patient information and discuss management plans with families [5,8].

Additionally, the use of complex medical terminology by healthcare professionals can confuse and alienate mothers, particularly those with low literacy levels or limited healthcare knowledge. Hesitation to ask questions, due to fear of judgment, perceived staff attitudes, or language barriers, further hinders effective communication. These challenges highlight the need for healthcare staff to adopt compassionate, clear, and culturally sensitive communication practices to support mothers in navigating the NICU experience [9].

The NICU environment, while focused on providing life-saving care for neonates, should also promote a family-centered care approach. This involves recognizing parents as integral members of the care team, empowering them with knowledge about their infant’s condition, and addressing their emotional and informational needs. Clear, timely, and empathetic communication fosters collaboration between parents and healthcare providers, reducing misunderstandings and promoting positive outcomes for both the infant and the family. Parents need to receive support and educational programs [10].

This study explores the communication difficulties faced by mothers of neonates admitted to NICUs and the impact of these challenges on their emotional well-being and engagement in their infant's care. By identifying barriers such as language difficulties, heavy workloads, and staff attitudes, the study aims to propose strategies for improving communication practices in NICUs. These include training healthcare staff to use simple, non-technical language, providing adequate time for parental interactions, and creating an environment of trust and empathy [11].

The findings from this research contribute to enhancing the quality of care in NICUs by addressing a critical yet often overlooked aspect of neonatal healthcare: effective communication. Improving parent-staff communication transforms the NICU experience for mothers, fosters confidence, reduces stress, and ultimately improves the overall care and outcomes for neonates.

## Materials and methods

A quantitative descriptive study design was used to assess the communication difficulties faced by mothers of neonates admitted to Neonatal Intensive Care Units (NICUs). Ethical committee approval was obtained from the Institutional Ethics Committee of Bharati Vidyapeeth College of Nursing (IECBVDUCONSANGLI - EC/NEW/INST/2024/MH/0414). The research was approved by the committee on April 8, 2024. Mothers of neonates who were admitted to the NICU for more than eight days and who were willing to provide written informed consent were included in the study. Mothers of critically ill neonates were excluded. The reliability of the data collection tool was assessed using the split-half method. A three-point scale with 16 items was used to collect data across four domains: communication difficulties related to staff approach, language, time, and environmental factors. The sample was identified with the help of the NICU in-charge. Participants were made comfortable, and the purpose of the study was explained prior to obtaining consent. Each participant completed the tool individually, and the process took approximately 15-20 minutes per participant.

Study participants

Study participants were selected using a nonprobability purposive sampling technique. A total of 80 mothers of neonates were selected for the study. After explaining the purpose of the study, written informed consent was obtained. The data collection tool was provided to the participants, and they were asked to complete it based on their experience in the hospital.

Statistical analysis

Data analysis was conducted using frequency and percentage. Baseline demographic variables were also analyzed using these methods. Communication difficulties were categorized into four domains: staff approach, language, time, and environmental factors. Each item under these domains was analyzed separately by calculating frequency and percentage. The analysis was performed using IBM SPSS Statistics for Windows, Version 24 (Released 2016; IBM Corp., Armonk, New York), and the results were presented in tables for better understanding.

## Results

Data collected from 80 participants were analyzed using appropriate statistical methods. Demographic variables were analyzed using frequency and percentage. Communication difficulties were analyzed under various categories, including communication difficulties related to staff approach, language, time, and environmental factors.

The baseline demographic variables of 80 participants included the mothers’ age, education, and duration of hospital stay. The majority of participants (50%) were aged 18-25 years, followed by 40% in the 26-33 years range, and a smaller proportion (10%) aged 34-40 years. In terms of education, 28.75% had primary education, 45% had completed secondary education, and 26.25% had higher secondary education. Regarding hospital stay duration, most participants (68.75%) stayed for 8-14 days, while 31.25% had hospital stays lasting 22-28 days. This information provides a clear demographic profile of the participants. The frequency and percentage distribution of age, education, and duration of hospital stay are shown in Table 1.

**Table 1:** Frequency and percentage distribution of age, education, and duration of hospital stay

S.No.	Variables		Category	Frequency (n)	Percentage (%)
1.	Mother’s age (in years)	18-25		40	50
		26-33		32	40
		34-40		8	10
2	Education	Primary		23	28.75
		Secondary		36	45
		Higher secondary		21	26.25
3	Duration of hospital stay (in days)	8-14		55	68.75
		22-28		25	31.25

The communication difficulties related to the staff approach were assessed among 80 participants. Most participants (86.25%) reported that staff always encouraged mothers to participate in caring for their child. Similarly, 72.5% felt that staff always spent adequate time caring for their child. However, only 37.5% stated that doubts about their child's condition were always cleared properly, and 48.75% said explanations about special treatments were always provided before procedures. Provision of written information and explanations about the functioning of machines used were always given to 30% and 55% of participants, respectively. This highlights varying levels of communication effectiveness across different aspects of staff interaction. The frequency and percentage distribution of communication difficulties related to staff approach are shown in Table 2.

**Table 2:** Frequency and percentage distribution of communication difficulties related to staff approach

S.No.	Statement	Always		Sometimes		Never	
		Frequency	Percentage (%)	Frequency	Percentage (%)	Frequency	Percentage (%)
1	Doubts regarding the child’s condition were cleared properly	30	37.5	40	50	10	12.5
2	Explanation regarding special treatment was given before the procedure	39	48.75	36	45	5	6.25
3	Provision of written information when needed	24	30	40	50	16	20
4	Explanation regarding the functioning of machines was given	44	55	23	28.75	13	16.25
5	Staff spent adequate time caring for your child	58	72.5	20	25	2	2.5
6	Encourage the mother to participate in caring for the child	69	86.25	11	13.75	0	0

The communication difficulties related to language were assessed among 80 participants. Half of the participants (50%) reported that the tone of language used by staff was always appropriate, while 25% felt it was sometimes appropriate, and the remaining 25% said it was never appropriate. The majority (76.25%) stated that the language used by staff was always easy to understand, while 18.75% found it sometimes easy, and 5% never found it easy to understand. Regarding the consistency of information, 66.25% reported that information was never varying or confusing, while 23.75% encountered some issues, and 10% always experienced it as confusing. This indicates that language was generally not a major barrier, though some inconsistencies in communication were noted. The frequency and percentage distribution of communication difficulties due to language are shown in Table 3.

**Table 3:** Frequency and percentage distribution of communication difficulties due to language

S.No.	Statement	Always		Sometimes		Never	
		Frequency	Percentage (%)	Frequency	Percentage (%)	Frequency	Percentage (%)
1	Tone of the language was appropriate	40	50	20	25	20	25
2	The language used by the staff was easy to understand	61	76.25	15	18.75	4	5
3	Information given by the staff was sometimes varying and confusing	8	10	19	23.75	53	66.25

Communication difficulties related to time were assessed among 80 participants. A majority (62.5%) reported that time was always given according to their needs, while 32.5% felt this was true only sometimes, and 5% felt it was never the case. Nearly all participants (95%) stated that visiting times were always feasible, with only 5% indicating this was sometimes true and none reporting it as never feasible. Regarding the timing of instructions, 31.25% felt they were always given at an appropriate time, while 68.75% said this was only sometimes the case. This suggests that while visiting times were well managed, there is a need for improvement in providing instructions at appropriate times. The frequency and percentage distribution of communication difficulties in relation to time are shown in Table 4.

**Table 4:** Frequency and percentage distribution of communication difficulties in relation with time

S.No.	Statement	Always		Sometimes		Never	
		Frequency	Percentage (%)	Frequency	Percentage (%)	Frequency	Percentage (%)
1	Time was given according to the person’s need	50	62.5	26	32.5	4	5
2	Feasible visiting time	76	95	4	5	0	0
3	Instructions were given during appropriate time	25	31.25	55	68.75	0	0

Communication difficulties caused by environmental factors were assessed among 80 participants. Emotional support was always provided when needed for 52.5% of participants, while 38.75% experienced this sometimes, and 8.75% reported never receiving it. A majority (57.5%) felt that staff were always sensitive to their emotions, while 28.75% felt this was true only sometimes, and 13.75% said it was never the case. The appropriateness of the counseling location was affirmed by 47.5%, with 41.25% finding it only sometimes appropriate, and 11.25% finding it never appropriate. While 55% reported always receiving enough time during counseling, 37.5% felt they never had sufficient time, and 7.5% reported this as sometimes true. These findings indicate variability in emotional and counseling support, with room for enhancing environmental factors to improve communication. The frequency and percentage distribution of communication difficulties due to environmental factors are shown in Table 5.

**Table 5:** Frequency and percentage distribution of communication difficulties due to environmental factors

S.No.	Statement	Always		Sometimes		Never	
		Frequency	Percentage (%)	Frequency	Percentage (%)	Frequency	Percentage (%)
1	Get’s emotional support whenever needed	42	52.5	31	38.75	7	8.75
2	Staff were sensitive to emotions	46	57.5	23	28.75	11	1.375
3	Place where counselling was given is appropriate	38	47.5	33	41.25	9	11.25
4	Enough time was given during counselling	44	55	6	7.5	30	37.5

## Discussion

The study was conducted to explore the communication difficulties faced by mothers of neonates admitted to NICUs. The experience of being separated from their newborns is an emotionally challenging time for any mother, often leading to feelings of stress, fear, and helplessness. Effective communication between healthcare providers and mothers is critical to alleviating these challenges and ensuring the well-being of both the mother and the neonate.

A qualitative study by Eva Karin Gotting and Ulrika Ferm highlighted the importance of proper communication between parents and NICU staff. Their research showed that providing adequate support and clear explanations reduced stress in mothers and minimized misunderstandings between them and healthcare workers [11]. Another study by Helena Wigert and Michaela Dellenmark examined parents' experiences of communication in NICUs and found that language barriers and the staff's approach often hindered effective communication. The study also revealed that parents’ emotional needs were frequently overlooked, further contributing to stress and dissatisfaction. These findings emphasize the unique role of healthcare workers in addressing parents' emotional difficulties and fostering open and supportive communication [3].

Similarly, a study by Jennifer Holditch-Davis and Susan O. Miles explored the psychological impact of preterm birth on mothers and found that a lack of emotional support from healthcare staff contributed to increased anxiety and depressive symptoms [12]. Their research indicated that mothers of preterm infants often experience heightened stress due to uncertainty about their child's survival and long-term health. The absence of empathetic communication and reassurance from NICU staff exacerbates these psychological challenges, leading to difficulties in mother-infant bonding and an increased risk of postpartum depression. The study underscored the importance of targeted mental health interventions and staff training programs to support maternal emotional well-being in NICU settings.

Another investigation by Jaeger and colleagues emphasized that the goal of baby- and family-centered care in the NICU is to recognize the baby's needs as exhibited through individual behavior and communication, and to support parent education, engagement, and interaction with the baby to build a nurturing relationship. Healthcare providers and caregivers must guide rather than control the role of the parents from birth through NICU care, transition to home, and continuing care at home. Parents are healthcare team members, primary caregivers, and shared decision-makers in caring for their babies [13]. Their study highlighted that structured parental involvement programs, including frequent updates, emotional counseling, and participatory decision-making, resulted in reduced stress and improved coping mechanisms among parents. They also found that parents who received personalized communication from healthcare providers felt more empowered and engaged in their infant’s care, reinforcing the importance of integrating family-centered approaches into NICU policies and practices.

The present study reinforces these findings, concluding that there is a significant need to prioritize the emotions of mothers and provide them with detailed explanations regarding their child’s condition and treatment modalities. By addressing these needs, healthcare workers can help reduce the emotional burden on mothers and build trust in the care process. Regular in-service education and training programs are essential to raise awareness among healthcare workers about their critical role in delivering quality care. Additionally, healthcare organizations should create opportunities to facilitate effective communication through proper staffing, training programs, and a supportive physical environment.

During the study, it became evident that mothers often hesitated to express negative feedback due to fear that it might affect the quality of care their child received. Similarly, healthcare workers were reluctant to communicate openly with mothers, fearing potential criticism that could impact their job security. These factors highlight the need for fostering an environment where both mothers and healthcare staff feel safe to communicate honestly and constructively.

The study faced several limitations. The use of structured statements in the data collection tool restricted participants from fully expressing their feelings, concerns, and insights. Incorporating interviews or open-ended questions could have allowed mothers to share their experiences in greater depth. Additionally, the study focused exclusively on mothers, leaving the perspectives of fathers and other family members unexplored.

To address these limitations, future research should adopt qualitative methods, such as in-depth interviews or focus groups, to gain a more comprehensive understanding of mothers’ experiences in NICUs. Studies should also explore the communication challenges faced by both parents and assess their perceptions of effective and ineffective communication practices. By addressing these gaps, future research can provide valuable insights to improve the quality of care and create a more inclusive and supportive NICU environment.

## Conclusions

The study concludes that there is a significant need to educate mothers about NICU processes, treatment procedures, and neonatal care to reduce their stress and help them feel more confident and involved in their child's care. When mothers are better informed about what is happening to their newborns and understand the care being provided, they are more likely to feel empowered and less anxious during this emotionally challenging time. This education not only benefits mothers but also fosters a stronger relationship between parents and healthcare providers, which can enhance the overall quality of care.

Additionally, healthcare workers should be trained to better understand the psychological state of parents, especially mothers, who often experience a range of emotions such as fear, anxiety, and frustration. Staff should be equipped with the skills to address these emotional needs with empathy and sensitivity, ensuring that parents feel heard and supported. Recognizing and addressing negative emotions in a compassionate manner is critical to building trust and reducing the risk of misunderstandings.

## Appendices

**Table 6:** Data collection tool

Data Collection Tool
Instructions
1. Kindly answer all questions
2. Please tick mark (✔) appropriate options
3. Collected data will be kept confidential
Code No:
Section A: Demographic variable
1. Age (years):
a. 18-25
b. 26-33
c. 34 and above
2. Education
a. Primary
b. Secondary
c. Higher secondary
3. Duration of hospital stay:

**Table 7:** Scale for assessing communication difficulties

S.No.	Statement	Always		Sometimes		Never	
		Frequency	Percentage (%)	Frequency	Percentage (%)	Frequency	Percentage (%)
	Approach of the staff						
1	Doubts regarding the child’s condition were cleared properly						
2	Explanation regarding special treatment was given before the procedure						
3	Provision of written information when needed						
4	An explanation regarding the functioning of machines was given						
5	Staff spent adequate time in caring for your child						
6	Encourage the mother to participate in caring for the child						
	Language						
7	The tone of the language was appropriate						
8	The language used by the staff was easy to understand						
9	Information given by the staff was sometimes varying and confusing						
	Time						
10	Time was given according to the person’s need						
11	Feasible visiting time						
12	Instructions were given at an appropriate time						
	Environmental factor						
13	Gets emotional support whenever needed.						
14	Staff were sensitive to emotions.						
15	The place where counselling was given is appropriate						
16	Enough time was given during counselling						
